# Hydrogen Sulfide (H_2_S)-Donor Molecules: Chemical, Biological, and Therapeutical Tools

**DOI:** 10.3390/ijms25147932

**Published:** 2024-07-20

**Authors:** Angela Corvino, Giuseppe Caliendo

**Affiliations:** Department of Pharmacy, School of Medicine, “Federico II” University of Naples, Via D. Montesano, 49, 80131 Naples, Italy; caliendo@unina.it

This Special Issue aims to gather new research on hydrogen sulfide (H_2_S)-releasing compounds (Figure 1) as cutting-edge pharmacological tools and to advance the understanding of the critical role that H_2_S plays in physiological and pathological processes.

Over the past two decades, H_2_S has emerged as the third recognized endogenous gasotransmitter [1,2] with several positive effects in different physiological and pathophysiological processes, such as inflammation, hypertension, oxidation, metabolic disorders, neuromodulation, and tumor progression [3,4,5].

Among the natural sources of H_2_S, there are polysulfides, which include garlic-derived compounds, and isothiocyanates, derived from vegetables belonging to the Brassicaceae family [6,7].

In addition to plants from the Alliaceae and Brassicaceae families, Citi and colleagues (contribution 1) performed a qualitative and quantitative analysis of the organosulfur compounds contained in mushroom extracts using an amperometric approach. Their studies confirmed the H_2_S-releasing capabilities of fungus extracts.

Moreover, the authors reported the pharmacological effects of natural H_2_S-donating compounds, including antioxidant properties, anti-inflammatory effects, regulation of the immune system, cardioprotection, and systemic metabolism’s regulation.

Interestingly, it has been demonstrated that natural isothiocyanates, including sulforaphane and erucin, significantly prevent and reduce the onset and spread of cancer [8]. In particular, several studies have proven the anti-tumor action of erucin (4-(methylthio)butyl isothiocyanate) [9].

Brancaleone et al., 2023 (contribution 2), investigate the anti-cancer properties of erucin in an in vitro model of triple-negative breast cancer. Erucin was found to significantly inhibit MDA-MB-231 cell proliferation by inducing apoptosis and autophagy in a concentration-dependent manner. Furthermore, the authors proved that erucin reduced intracellular ROS generation, boosting the activation of critical antioxidant genes and stopping MDA-MB-231 cell movement, invasion, and colony formation.

Moreover, to further define the physio-pathological functions of H_2_S, synthetic H_2_S-donating compounds have been developed.

In this regard, 4-methoxy-phenyl(morpholino)phosphinodithioate morpholinium salt, named GYY4137, is the earliest synthesized and characterized H_2_S donor molecule [10]. Due to its capability to gradually release H_2_S, it is the most widely studied H_2_S donor to elucidate the involvement of H_2_S signaling pathway in specific physio-pathological conditions [11,12,13,14].

More recently, Berenyiova and colleagues (contribution 3), through the intraperitoneal application of GYY-4137, investigated the involvement of NO and H_2_S pathways in fructose-fed spontaneously hypertensive rats with metabolic disorders. They found that the slow H_2_S-releasing donor activated the endogenous sulfide pathway in the thoracic aorta, with a consequent positive pro-relaxant and anti-contractile effect.

Ishkaeva et al., 2023 (contribution 4), develop novel synthetic H_2_S donating molecules. These compounds are derived from the combination of glutathione with different H_2_S donors, dithiophosphates.

The kinetics of H_2_S generation from the new compounds indicated that the donors were continuously releasing H_2_S, with the dithiophosphate of reduced glutathione releasing more H_2_S than oxidized glutathione. The compounds that effectively increased intracellular H_2_S prevented C2C12 myoblasts from proliferating at submillimolar doses, in contrast to NaHS. Overall, these studies revealed glutathione dithiophosphates as redox-modulating H_2_S donors with a long-acting profile.

As well as being used as pharmacological tools for further studies on the functions of H_2_S in the body, some of the H_2_S-releasing moieties have been largely combined with different drugs already used in the clinic to obtain novel multi-target molecular hybrids [15].

In the study by Sparaco et al., 2022 (contribution 5), new molecular hybrids between antiglaucoma drugs, such as brinzolamide, betaxolol, and brimonidine, and H_2_S donors were designed and synthesized.

All new compounds were proven to release H_2_S both in aqueous solutions and in the intracellular environment of human primary corneal epithelial cells. Moreover, their findings indicated two brinzolamide derivatives and one brimonidine H_2_S donor as the best hybrids, characterized by a significant and long-lasting production of the gasotransmitter. Their findings allow for more intensive glaucoma therapy to be provided.

## Figures and Tables

**Figure 1:**
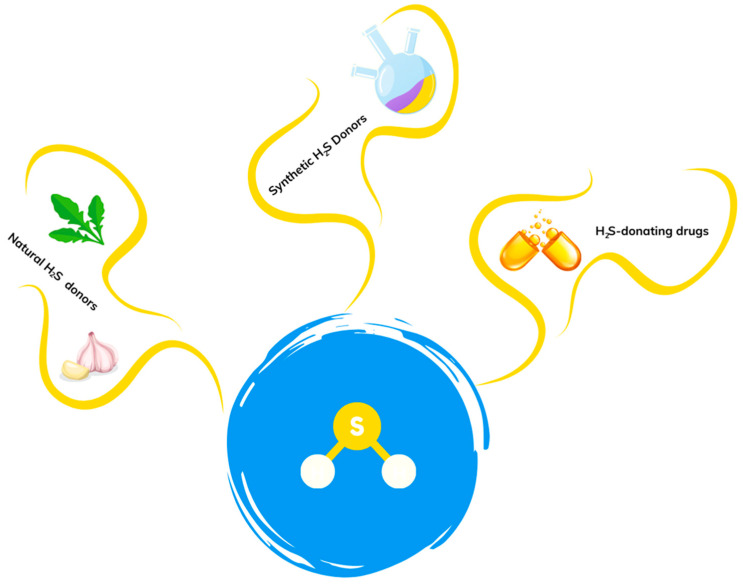
Different sources of H_2_S: natural and synthetic H_2_S donors, and H_2_S-donating drugs.
